# Financial capability and economic well-being of older Americans: empirical insights from the financial capability framework

**DOI:** 10.1093/geroni/igag003

**Published:** 2026-01-10

**Authors:** Yu-Chih Chen, Sicong Sun, Jin Huang, Margaret S Sherraden

**Affiliations:** Department of Social Work, National Taiwan University, Taipei, Taiwan; Department of Social Welfare, Luskin School of Public Affairs, University of California, Los Angeles, Los Angeles, California, United States; Brown School, Washington University in St Louis, St Louis, Missouri, United States; Brown School, Washington University in St Louis, St Louis, Missouri, United States

**Keywords:** Economic security, Financial well-being, National Financial Capability Study, Age difference, Life course perspective

## Abstract

**Background and Objectives:**

Financial capability, the interaction of financial literacy, access, and behavior, can influence individuals’ ability to manage finances and build economic stability over the life course. However, empirical evidence linking financial capability and economic well-being in old age is limited. We examine the components and mechanisms of the financial capability framework and investigate the differences between middle-aged (aged 50–64) and older adults (aged 65+) using population-based data in the United States.

**Research Design and Methods:**

The respondents, 12,840 individuals aged 50 and over, were selected from the population-based 2018 National Financial Capability Study. Structural equation modeling and multigroup analyses were used to examine the mechanisms of financial capability on economic well-being and the moderating effects of age.

**Results:**

Financial literacy and access are positively associated with financial behavior and subsequent economic well-being. The mediation results show that financial literacy and access are equally important in contributing to economic well-being in later life. However, age has no moderating effect, suggesting these associations operate similarly in middle-aged and older adults.

**Discussion and Implications:**

Strategies to promote economic well-being in later life through effective finance should be multifaceted, targeting each aspect of financial capability. Financial practices such as financial coaching and guidance, credit counseling, and financial assessments should be integrated into social services. Policy innovations that expand financial inclusion, particularly investments in accessible and appropriate financial products, should be developed to reach and cover the financially underserved, especially those with limited financial literacy and restricted access to mainstream financial services.

Innovation and Translational Significance: This study advances understanding of financial capability and its influence on the economic well-being of middle-aged and older adults. The findings highlight the equal importance of financial literacy and financial inclusion in shaping financial behavior and promoting economic stability in individuals aged 50+. Notably, the mechanisms linking financial capability to economic well-being are consistent across age groups, indicating similar pathways for both middle-aged and older adults. These results underscore the need for integrated, age-inclusive strategies such as financial coaching, credit counseling, and inclusive financial products to strengthen all dimensions of financial capability and promote economic security in later life.

Older Americans are at risk of falling into economic insecurity (National Council on Aging, 2024a). Recent estimates show that more than 17 million older Americans aged 65 and older are economically insecure, with incomes below 200% of the Federal Poverty Line (National Council on Aging, 2024b). Additionally, among older households aged 65 and older, 61% held debt with a median value of $31,050 (Li, 2019); and nearly half of adults aged 55–74 had no retirement savings (National Council on Aging, 2024b). Emerging research further shows that the economic well-being of older Americans during the pandemic era did not move in a single direction. Using the Supplemental Poverty Measure—which adds tax credits and in-kind benefits (e.g., SNAP) and subtracts taxes and medical out-of-pocket costs—poverty among older adults fell from 2019 to 2020 with federal relief. Among older adults, it then increased from 2020 to 2021, and again in 2022. Over 2020–2021, food insecurity among older adults increased by about 8% (Ochieng et al., 2024; United Health Foundation, 2024).

Financial capability—encompassing various components including financial knowledge and skill (i.e., financial literacy), prudent behavior (i.e., financial behavior), and access to financial services, programs, and policies (i.e., financial access and inclusion)—may enhance economic well-being via making sound saving decisions, effective use of financial products and services, setting financial goals, and adhering to a budget (Consumer Financial Protection Bureau, 2018). Despite the growing relevance of financial capability for the aging population, the empirical links between financial capability and the economic well-being of older adults remain understudied. Moreover, it is unclear if these associations vary across different age groups of older adults. Using a structural equation modeling (SEM) approach, this study aims to address these gaps by (1) distinguishing among the three components of financial capability; (2) testing financial behavior as a mediator linking financial literacy, financial access, and economic well-being; and (3) exploring age group variations in these pathways.

By examining these mechanisms, this study provides insights into strategies to enhance economic stability among older adults through key components of financial capability, including bridging gaps in financial knowledge, fostering financial inclusion, and promoting informed planning. Advancing economic well-being across the life course through these targeted efforts can help build more inclusive financial systems that are responsive to the challenges faced by older adults, which ultimately improve both individual well-being and overall societal outcomes.

## Conceptualization of financial capability

Building on Amartya Sen’s (1993) capability theory, which emphasizes both internal capacity and external conditions, financial capability captures the idea that economic well-being results from the interaction of individuals’ ability to act with their access to financial structures and opportunities (Birkenmaier et al., 2022; Sherraden, 2013). Sherraden (2013) further defines financial capability as a multidimensional framework encompassing financial literacy, access, and behavior that enables people to act in their best financial interest. Financial literacy indicates an individual’s ability to make informed financial decisions (Xue et al., 2021), which refers to numeracy skills, knowledge of financial concepts and risks, confidence, and self-efficacy (Fernandes et al., 2014). Financial literacy is typically measured objectively (e.g., correct understanding of financial issues) and subjectively (e.g., ability to manage financial matters) (Goyal & Kumar, 2021). Financial access represents one’s opportunity to act, which is usually measured by one’s level of financial inclusion (Birkenmaier et al., 2019), such as accessibility to mainstream financial policies, products, and services (e.g., bank accounts, savings and investments, insurance, and credit) that meet one’s needs (Birkenmaier et al., 2022). However, variations in financial access may result from varied power, resources, or knowledge due to individual differences in age, gender, race, education, or financial situation (Sherraden et al., 2018). Lastly, financial behavior pertains to actions and behaviors that align with one’s best financial interests (Birkenmaier et al., 2022), which refers to sound financial management practices in dealing with financial matters (Sun et al., 2022; Xiao & O’Neill, 2018). It is typically measured by practices such as setting and achieving financial goals, intention or propensity to save, and budgeting and planning.

The financial capability framework by Sherraden (2013) posits a sequential and dynamic process in which financial literacy and access affect financial actions, leading to positive economic well-being and enhanced financial development. A growing body of research has applied this framework to examine financial capability in general or younger populations. For instance, Despard et al. (2020) examined the associations between financial literacy (both objective and subjective), financial access (measured by savings account ownership), and setting aside emergency funds among U.S. households aged 18 and older. Friedline and West (2016) investigated whether financial capability (combined with financial education and savings account ownership) is associated with economic well-being (e.g., financial satisfaction, emergency fund, or debt burden) among 6,865 U.S. millennials aged 18–34. Lastly, Fu (2020) examined how financial access (measured by saving, credit, and insurance products), financial knowledge (e.g., compound interest, risk and return, inflation), and financial behavior (e.g., budgeting, saving, timely payment) affect economic well-being (e.g., balancing income and expense, planning, managing debts) among individuals aged 16 and older from 11 OECD economies.

However, these studies only examine one or a few components of the financial capability framework, or they use a single item to measure components of financial capability. Furthermore, these studies examine only the independent effects of selected indicators within the financial capability framework without testing the framework’s systematic components and sequential relationships. An exception is a recent study by Sun et al. (2022), which used a sample of 24,154 US households aged 18 and older to examine the relationship between financial capability and economic hardship. They found that financial literacy (subjective and objective) and financial access (measured by banking accounts, investment accounts, and credit cards) were positively associated with financial behavior (e.g., setting long-term goals) and subsequently reduced economic hardships (e.g., difficulty covering expenses and medical hardships).

Above all, this framework has received little attention in population-based studies of older adults, and findings remain inconclusive. Some studies have applied the financial capability framework using convenience samples or in studies of marginalized respondents. For instance, using an online survey of 1,109 adults aged 45 and older in Hong Kong, Chen and Sun (2023) found that financial access and behavior have a greater effect on health than financial literacy. Additional evidence comes from studies of low-income older Asian immigrants (*n *= 142–159) in the Senior Community Service Employment Program in Los Angeles and New York City (Huang et al., 2015; Nam et al., 2016). These studies found that financial access and behavior are negatively associated with economic hardship, whereas the effect of financial literacy is not significant. Lastly, Nam and Loibl (2021) used a non-retired, low-income sample of 981 adults aged 55 and older. They found that financial access (measured by savings account ownership) has a stronger association with financial behaviors (e.g., saving for retirement or being able to cover expenses) than financial education. These mixed findings, based on context-specific samples, highlight the need for comprehensive investigations into the dynamic nature and applicability of the financial capability framework in population-based older adults.

## Financial capability and economic well-being

Empirical research has shown that financial capability is positively correlated with indicators of economic well-being. Evidence includes increased wealth or savings for retirement (Hastings & Mitchell, 2020; Lusardi & Mitchell, 2007a; Nam & Loibl, 2021), retirement planning or actions (Lusardi & Mitchell, 2007a, 2011), setting aside rainy-day or emergency funds (Despard et al., 2020; Friedline & West, 2016; Nam & Loibl, 2021; Xue et al., 2021), higher satisfaction with current finances in terms of income or debts (Adam et al., 2017; Friedline & West, 2016; Xiao & O’Neill, 2018; Xiao & Porto, 2017), lower retirement anxiety and material hardships (Huang et al., 2015; Kadoya et al., 2018), and the ability to cover expenses (Fu, 2020; Nam & Loibl, 2021; Xue et al., 2021).

However, there are research gaps. Although financial capability has been widely studied across disciplines, its measurement lacks consistency due to variations in item selection and indicator combinations, often driven by researchers’ discretion or data constraints (Chen & Sun, 2023). Several reviews and meta-analyses have shown that current research tends to focus narrowly on individual components—most commonly financial literacy or behavior—rather than treating financial capability as a multidimensional construct (Goyal & Kumar, 2021; Santini et al., 2019). Notable exceptions include studies by Sun et al. (2022) and Huang et al. (2015), but these focus on households overall, or specifically on low-income older adults. Furthermore, because many existing studies rely on regression-based analyses, they are limited in capturing the dynamic, multidimensional nature of financial capability. More holistic research is needed to examine the full scope of financial capability—literacy, access, and behavior—and the mechanisms that link these components. Given the inconsistencies in measurement and the lack of empirical testing of the theoretical sequence, further research is warranted to clarify how these elements interact and shape economic outcomes, especially among older adult populations.

## The moderating effect of age

Evidence suggests there are age-related variations across components of financial capability. When comparing older adults (e.g., age 55 and above) with younger populations, for example, age is positively associated with objective financial literacy, subjective financial literacy, desirable financial behaviors, and greater financial satisfaction (Xiao et al., 2015; Xiao & Porto, 2017). However, studies that focus solely on older adults reveal a more nuanced pattern. For example, although subjective financial literacy (e.g., confidence in making financial decisions) tends to remain stable with age, objective financial literacy declines, likely due to age-related cognitive decline (Finke et al., 2017; Gamble et al., 2015). Similarly, a recent national survey found that adults aged 50–64 experience more financial challenges than those aged 65 and older across multiple indicators of financial well-being (Kullgren et al., 2024). Financial strain before age 65 complicates retirement planning and may lead to greater financial insecurity later in life, as income typically decreases after retirement (Chen & Zurlo, 2022; Kullgren et al., 2024). These findings suggest that the relationship between age and each component of financial capability remains inconclusive and merits further investigation. However, existing research often treats age merely as a control variable (Xiao et al., 2015), rather than examining its moderating role in the relationship between financial capability and economic well-being.

As some of these examples demonstrate, economic well-being in later life is shaped in large part by financial capability accumulated across the life course. Financial capability develops through a series of financial experiences and decisions, influenced by the historical periods and institutional contexts in which individuals live (Morrow-Howell & Sherraden, 2015). Drawing on the life course perspective (Elder et al., 2003), Morrow-Howell and Sherraden (2015) propose that several factors shape financial capability in later life. It is affected by the socioeconomic conditions and historical events (e.g., recessions or policy changes) that occur during a person’s lifetime. Financial capability is also relational as it is influenced by social relationships (e.g., socialization and social support). Individuals’ financial choices and actions are influenced by the opportunities and constraints present across the life course, which create lasting advantages or disadvantages in financial capability.

Guided by the life course perspective, variations in financial capability resulting from these life course mechanisms could be observed through age differences. For instance, individuals born at different times and in different cohorts may have distinct experiences with financial institutions and opportunities for financial engagement. As a result, their financial behaviors and attitudes are shaped by varying sociocultural and structural contexts (Morrow-Howell & Sherraden, 2015). Additionally, financial socialization and guidance from others may influence financial decision-making and development (Sun et al., 2022). Building on the life course perspective and current research, we propose that age-related differences in financial capability may translate into disparities in economic well-being. Given the lack of clear evidence on the direction and strength of the relationship between financial capability and economic well-being across age groups, we hypothesize that age moderates these associations.

## This study

Applying the financial capability framework, this study examines how financial literacy and access shape financial behavior and how these, in turn, influence economic well-being in later life. Grounded in a life course perspective, we explore whether these relationships differ by age among older adults. To move beyond context-specific findings based on low-income or convenience samples, we draw on a population-based U.S. dataset: the 2018 National Financial Capability Study. Specifically, structural equation modeling (SEM) is employed to test the proposed sequence of pathways. Compared to regression-based approaches, SEM provides a more comprehensive analysis by modeling all components simultaneously and capturing both direct and indirect effects. We also conduct multigroup comparisons to assess whether the associations vary across age groups, ensuring that differences reflect group variation rather than inconsistencies in measurement. We propose the following hypotheses:

H1: Financial literacy and financial access are positively associated with financial behavior.

H2: Financial behavior mediates the relationships between financial literacy, financial access, and economic well-being.

H3: Age moderates the associations between financial capability and economic well-being.

## Methods

### Data and sample

We used the National Financial Capability Study (NFCS) conducted by the Financial Industry Regulatory Authority Investor Education Foundation. The NFCS is a repeated cross-sectional triennial survey, initiated in 2009, that collects extensive variables in financial literacy, access, and behavior, with follow-up surveys conducted from 2012 to 2024. Survey measures vary slightly across waves. The NFCS surveys a nationally representative sample of approximately 27,000 individuals (approximately 500 respondents per state) aged 18 and older, with oversampling in New York, Texas, Illinois, and California. The sampling mirrors the U.S. Census distributions for age, gender, ethnicity, education level, and income within states, provided that all individuals are analyzed with a weight. However, analyses of subpopulations (e.g., individuals of a specific age group) may not be representative. We selected the 2018 wave of NFCS because it includes comprehensive measures of the three components of financial capability (literacy, access, and behavior) and economic well-being. Moreover, its large, population-based sample provides sufficient statistical power to test the three proposed hypotheses. We restricted the analytic sample to respondents aged 50 and older (*n *= 12,840) as this study examines financial capability in later life.

### Measures

*Financial capability.* Guided by the financial capability framework, we constructed three latent factors for financial literacy, access, and behavior. Using the methods developed by Sun et al. (2022), financial literacy was measured by three ordinal indicators that capture both subjective (e.g., confidence and self-efficacy in financial matters) and objective (e.g., numeracy skills and knowledge of financial concepts) aspects of financial literacy. Subjective financial literacy was assessed by two variables: self-perceived financial knowledge (1 = *low*; 7 = *high*) and whether good at dealing with daily financial matters (1 = *strongly agree*; 7 = *strongly disagree*). Objective literacy was assessed by the sum of correct responses in six financial literacy questions on interest rates, mortgages, bonds, stocks, and inflation (*range*: 0–6; see Supplementary Table 1 for detailed item description). The original objective literacy questions were developed by Lusardi and Mitchell (2007b) and have since been widely used to assess financial knowledge. Financial access was a latent variable constructed based on whether respondents had the following financial products (0 = *no*; 1 = *yes*): checking accounts, savings accounts, investment accounts, individual retirement accounts, and credit cards. Lastly, financial behavior was measured by two binary measures related to prudent financial management and practice: setting emergency or rainy-day funds and not overspending their household income.

*Economic well-being.* Following previous research (Friedline & West, 2016), the latent economic well-being was measured by four variables: self-rated financial satisfaction (1 = *not satisfied*; 10 = *very satisfied*), reversed-coded retirement worry (1 = *very worry*; 7 = *not worry*), self-rated on the statement “I am just getting by financially” (1 = *agree*; 5 = *disagree*), and confidence in coming up with $2,000 in an emergency (1 = *certainly cannot*; 4 = *certainly can*).

*Covariates.* Following prior research on determinants of financial capability and economic well-being (Goyal & Kumar, 2021; Santini et al., 2019), we controlled for covariates that were statistically significant in the SEM models (see Analyses section for details about the SEM): whether received financial education, age (continuous), gender (1 = *women*; 0 = *men*), education levels (1 = *no high school degree*; 7 = *postgraduate*), income (1 = *<$15K*; 8 = *$150K+*), and race (1 = *white*; 0 = *non-white*). Race was coded as a binary variable because non-white subgroups (Black, Hispanic/Latino, Asian, other/multiple) had small cell sizes. We also tested marital status, employment, number of children, and health insurance in preliminary models, but these were not significantly related to financial capability or economic well-being. Following recommendations to avoid including weak predictors that increase model complexity (Hair et al., 2019), these variables were excluded to maintain model parsimony.

### Analyses

Descriptive and bivariate analyses were used to examine the distributions of covariates and financial capability indicators by total and age groups (50–64 and 65+). We used a structural equation modeling (SEM) approach to examine the financial capability framework, in that how financial literacy and access were associated with financial behavior (H1) and whether financial behavior mediated the relationships between financial literacy, financial access, and economic well-being (H2). We further examined whether age moderated the links between the components of financial capability and economic well-being using multigroup analysis (H3). We used SEM because of its advantages in testing theoretically driven models that involve multiple relationships (direct and mediating effects) among numerous variables simultaneously and in providing model fit indices for assessing the goodness of fit of the models for both cross-sectional and panel data (Hair et al., 2019; Wang & Wang, 2019). The SEM included both a measurement model to test the fit of the proposed latent factor structures and a structural model to examine the associations (or paths) among the latent factors. Both measurement and structural models were applied to the whole sample and to two age groups (aged 50–64 and 65+) to test the proposed hypotheses. The steps of the SEM analyses were presented as follows.

First, a measurement model using confirmatory factor analysis (CFA) was conducted to examine the fit of latent constructs of financial literacy, access, behavior, and economic well-being. We then used a structural path model to examine the direct and indirect paths linking the three constructs of financial capability and economic well-being in the total sample (H1 and H2). To further explore the differences between the two indirect paths (financial literacy and financial access in predicting financial behavior and economic well-being), the model constraint analysis using the Wald test was employed (Wang & Wang, 2019). These analyses were all conducted based on all respondents.

To test H3—the age-group moderation (50–64 vs 65+) of links between financial capability and economic well-being—we must first establish measurement invariance; only then can differences be attributed to path coefficients rather than to measurement. We conducted a series of measurements and structural invariance models following the methods developed by Byrne (2013). We first examined the CFA model separately for two age groups to assess whether the proposed latent structure provided a good fit. Next, we examined the multigroup measurement invariance analyses using a three-step sequential model. The first step began with a configural invariance model, in which each item is associated with the same latent factor across groups, with all parameters freely estimated across groups. A satisfactory configural invariance model indicates that similar latent constructs were measured across groups and serves as a baseline for evaluating goodness-of-fit against a series of increasingly restrictive models. The second step was the metric invariance model, in which the factor loadings were constrained to be equal across groups. The last model was the scalar model, in which the intercept or threshold of each item was constrained to be equal across groups. The metric invariance model should be satisfied to ensure proper comparisons in structural paths, particularly in the moderation hypothesis that requires comparing relationships across groups (Hair et al., 2019). The model differences were evaluated using the chi-square difference test; an insignificant chi-square difference indicated that the targeted measurement (factor structure, loadings, and intercepts or thresholds) was invariant across the two groups (Hair et al., 2019).

After establishing measurement invariance, we then conducted a multigroup structural path model to examine whether age moderates the associations between financial capability and economic well-being. The baseline model constrained factor loadings to be equal, but structural paths were freely estimated across the two groups. Then, a fully constrained model was estimated in which all loadings and structural paths were constrained to be equal across groups. These two models were compared using the chi-square difference test, and a significant chi-square value indicated the moderating effect of age.

We applied a bias-corrected bootstrapped method with 5,000 resamplings to calculate the indirect effects, and significance was presented with the 95% confidence interval (CI) (MacKinnon et al., 2002). We used the weighted least squares mean and variance adjusted (WLSMV) estimator for both measurement and structural models to account for the binary or ordinal nature of the variables. Approximately 14% of respondents were missing in financial education and other variables. We compared the results with and without multiple imputations (*n *= 20; see Supplementary Table 2), and the results were almost identical. Thus, we used listwise deletion as bootstrapped indirect effects could not be obtained in multiple imputations.

Following Wang and Wang (2019), the comparative fit index (CFI), Tucker-Lewis index (TLI), and root mean square error of approximation (RMSEA) with a 90% CI were used to assess the model fits of SEM. The model chi-square was not used for assessing model fit due to its sensitivity to larger sample sizes (Wang & Wang, 2019). A good model fit for an SEM model was indicated by CFI and TLI >0.90 and RMSEA <0.05, with an upper bound of 90% CI <0.08 (Hu & Bentler, 1999). As this study used a subsample of individuals aged 50+, the analyses were unweighted because the weights were only applicable when all respondents were included. The SEM analyses were conducted using M*plus* 8.11, and others using Stata 17.0.

## Results

### Sample characteristics

Table 1 summarizes the sample characteristics and comparisons by age group. The mean age of all respondents was 62.97. Among all respondents, gender was distributed almost equally (women; 54.21%), the majority was White (84.10%), 36% had at least some college degree, and approximately 60% had an income of more than $50,000. Approximately one-fifth reported receiving financial education. Results of age group comparisons showed that the older group (i.e., 65+) was predominantly white (88.37%), had slightly higher education, and had lower rates of receiving financial education than those aged 50–64. Components of financial capability also vary significantly by age group (see Supplementary Table 1). Older groups tend to score higher in financial literacy, financial access, financial behavior, and financial satisfaction and confidence in coming up with $2,000. However, they also reported higher retirement worries and were more likely to get by financially.

**Table 1.** Sample characteristics and comparison by age group (aged 50–64 and aged 65+).

Covariates	All (*N *= 12,840)	Age 50–64 (*n *= 7,328)	Age 65+ (*n *= 5,512)	*χ^2^/t*
**Age (years)**	62.97 (8.10)	57.16 (4.33)	70.68 (4.85)	−177***
**Gender**				2.73
** Women**	6,960 (54.21%)	3,926 (53.58%)	3,034 (55.04%)	
** Men**	5,880 (45.79%)	3,402 (46.62%)	2,478 (44.96%)	
**Race**				131.93***
** White**	10,798 (84.10%)	5,927 (80.88%)	4,871 (88.37%)	
** Non-White**	2,042 (15.90%)	1,401 (19.12%)	641 (11.63%)	
**Education**				106.60***
** Less than high school**	221 (1.64%)	142 (1.94%)	69 (1.25%)	
** High school**	2,506 (19.52%)	1,459 (19.91%)	1,047 (19.52%)	
** GED**	867 (6.75%)	501 (6.84%)	366 (6.64%)	
** Some college**	3,270 (25.47%)	1,904 (25.98%)	1,366 (24.78%)	
** Associated degree**	1,362 (10.61%)	882 (12.04%)	480 (8.71%)	
** Bachelor**	2,694 (21.98%)	1,507 (20.56%)	1,187 (21.53%)	
** Post-graduate**	1,930 (15.03%)	933 (12.73%)	997 (18.09%)	
**Household income**				129.66**
** Less than $15,000**	981 (7.64%)	660 (9.01%)	321 (5.82%)	
** $15,000–$24,999**	1,273 (9.91%)	710 (9.69%)	563 (10.22%)	
** $25,000–$34,999**	1,380 (10.75%)	721 (9.84%)	659 (11.96%)	
** $35,000–$49,999**	1,828 (14.24%)	951 (12.98%)	877 (15.91%)	
** $50,000–$74,999**	2,627 (24.46%)	1,404 (19.16%)	1,223 (22.19%)	
** $75,000–$99,999**	1,865 (14.52%)	1,067 (14.56%)	798 (14.48%)	
** $100,000–$149,999**	1,831 (14.26%)	1,139 (15.54%)	692 (12.55%)	
** $150,000 or more**	1,055 (8.22%)	676 (9.22%)	379 (6.88%)	
**Received financial education**	2,079 (18.99%)	1,255 (20.04%)	821 (17.59%)	10.41***

*Note.* GED = General Educational Development. Mean (standard deviation) and *t* are reported for age; *N* (%) and *χ^2^* are reported for all other covariates (i.e., categorical variables).

* *p* < .05.

** *p* < .01.

*** *p* < .001.

### Measurement model

The CFA model results (see Table 2) suggested a satisfactory model fit (*χ^2^*_(__*71*__)_ = 3,705.456, *p* < .001; *CFI *= 0.961; *TLI *= 0.950; *RMSEA *= 0.063 [90% CI: 0.061–0.065]), and all standardized factor loadings were significant (*p* < .001) and above 0.40. The correlation across the four latent factors was significant at the 0.001 level. Financial literacy was positively correlated with financial access (*r *= 0.67, *p* <.001). Financial access (FA; *r *= 0.85, *p* < .001) showed a stronger correlation with financial behavior (FB) than financial literacy (FL; *r *= 0.69, *p* < .001). Lastly, financial behavior (*r *= 0.91, *p* < .001) had a stronger correlation with economic well-being (EW) than financial literacy (*r *= 0.72, *p* < .001) and financial access (*r *= 0.79, *p* < .001).

**Table 2.** Measurement model results.

Latent variables	Items	Estimates (*λ*/*r*)
**Financial literacy (FL)**	(L1) Day-to-day financial matters	0.522***
	**(L2) Financial knowledge**	0.646***
	**(L3) Financial literacy questions**	0.534***
**Financial access (FA)**	(A1) Checking account	0.716***
	**(A2) Savings account**	0.748***
	**(A3) Individual retirement account**	0.871***
	**(A4) Investment account**	0.818***
	**(A5) Have credit card**	0.777***
**Financial behavior (FB)**	(B1) Rainy-day fund	0.936***
	**(B2) Spend less or equal to household income**	0.407***
**Economic well-being (EW)**	(E1) Financial satisfaction	0.773***
	**(E2) Retirement worry**	0.554***
	**(E3) Getting by financially**	0.810***
	**(E4) Confidence in coming up with $2,000**	0.914***
***Correlation***		
** FL ⟷ FA**		0.673***
** FL ⟷ FB**		0.691***
** FA ⟷ FB**		0.848***
** FL ⟷ EW**		0.722***
** FA ⟷ EW**		0.798***
** FB ⟷ EW**		0.916***

*Note.*  *CFI* = Comparative Fit Index; *TLI* = Tucker-Lewis Index; *RMSEA* = Root Mean Square Error of Approximation; *λ* = item factor loading (standardized) for latent variables, and all factor loadings were significant; *r* = standardized correlation. Results were estimated using weighted least squares to correct the categorical nature of indicators. Model fit: *χ^2^*_(__*71*__)_ = 3,705.456, *p* < .001; *CFI * =  0.961; *TLI *= 0.950; *RMSEA *= 0.063 (90% CI: 0.061, 0.065).

*** *p* < .001.

### Structural path model

**Figure 1.**
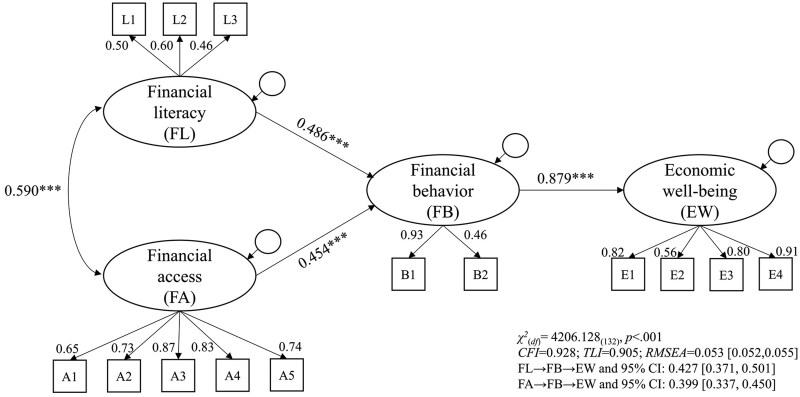
Structural equation modeling for direct and indirect effects between financial capability and economic well-being (standardized estimates). Note. The indirect effects were estimated with 5,000 bootstraps. The effects of covariates and item residuals were not presented.

Figure 1 shows the structural path model for the association between financial capability and economic well-being, controlling for covariates. The results (see Table 3) showed an acceptable model fit (*χ^2^*_(__*132*__)_ = 4,206.128, *p* < .001; *CFI *= 0.928; *TLI *= 0.905; *RMSEA *= 0.053 [90% CI: 0.052–0.055]), and all the paths were significant at the 0.001 level. The standardized path estimates showed that financial literacy (*β *= 0.486) and financial access (*β *= 0.454) were positively correlated with financial behavior, with financial literacy demonstrating a slightly stronger association with financial behavior than financial access. In addition, financial behavior was positively associated with economic well-being (*β *= 0.879). The indirect effects with 5,000 bootstrap procedures (see Table 3) yield significant results (FL→FB→EW: 0.427 [90% CI: 0.371, 0.501]; FA→FB→EW: 0.399 [90% CI: 0.337, 0.450]), suggesting that financial behavior mediated the relationship between financial literacy, financial access, and economic well-being. As the indirect effect of financial literacy appears to be stronger than that of financial access, we conducted parameter constraints to test the differences in these two indirect effects. The Wald test results showed that the comparison was insignificant (*χ^2^_(1)_* = 1.10, *p *> 0.05), suggesting similar indirect effects. Regarding covariates, the results (see Supplementary Table 3) showed that older individuals and those with higher incomes, as well as those who had previously received financial education, reported higher levels of financial literacy, financial access, and economic well-being. In contrast, women, being white, and those with higher education levels reported lower economic well-being.

**Table 3.** Path of direct and indirect effects with 5,000 bootstrap procedures.

Paths	*b/β*	*SE*	95% CI
**Direct effects (unstandardized *b*)**			
** FL→FB**	0.810***	(0.072)	
** FA→FB**	0.696***	(0.065)	
** FB→EW**	1.838***	(0.038)	
**Indirect effects (unstandardized *b*)** ^a^			
** FL→FB→EW**	1.489***	(0.133)	[1.267, 1.786]
** FA→FB→EW**	1.280***	(0.119)	[1.054, 1.513]
**Direct effects (standardized *β*)**			
** FL→FB**	0.486***	(0.037)	
** FA→FB**	0.454***	(0.033)	
** FB→EW**	0.879***	(0.012)	
**Indirect effects (standardized *β*)** ^a^			
** FL→FB→EW**	0.427***	(0.033)	[0.371, 0.501]
** FA→FB→EW**	0.399***	(0.029)	[0.337, 0.450]
**Model fits**			
***χ^2^_(df)_***	4,206.128***_(132)_		
***CFI***	0.928		
***TLI***	0.905		
***RMSEA* [90% CI]**	0.053 [0.052, 0.055]		

*Note.* FL = financial literacy; FA = financial access; FB = financial behavior; EW = economic well-being; *SE* = standard error; CI = confidence interval; CFI = Comparative Fit Index; TLI = Tucker-Lewis Index; RMSEA = Root Mean Square Error of Approximation. The estimates were based on all respondents as no group differences were found via multigroup analysis; all analyses were controlled for the covariates listed in Table 1.

a The Wald test for parameter constraints for indirect effects was insignificant (*χ^2^_(1)_* = 1.10, *p *> 0.05).

*** *p* < .001.

### Multigroup analysis

The test of age differences in the structural paths of financial capability to economic well-being (i.e., multigroup structural invariance) should be based on a similar measurement model across two age groups (i.e., multigroup measurement invariance). The findings of the measurement invariance (see Supplementary Table 4) showed that the measurement model demonstrated good fit in each age group (aged 50–64: *χ^2^*_(__*71*__)_ = 2,175.294, *p* < .001; *CFI *= 0.960; *TLI *= 0.948; *RMSEA *= 0.064 [90% CI: 0.061–0.066]; aged 65+: *χ^2^*_(__*71*__)_ = 1,718.474, *p* < .001; *CFI *= 0.953; *TLI *= 0.940; *RMSEA *= 0.065 [90% CI: 0.062–0.068]), and both groups demonstrated the same latent factor structure with acceptable model fit when estimated combined (*χ^2^*_(__*142*__)_ = 3,798.249, *p* < .001; *CFI *= 0.945; *TLI *= 0.929; *RMSEA *= 0.063 [90% CI: 0.062–0.065]), suggesting the configural model was established. We further examined the metrics model assuming the same factor loadings across the two groups, and the test yielded good model fit (*χ^2^*_(__*152*__)_ = 2,622.736, *p* < .001; *CFI *= 0.963; *TLI *= 0.954; *RMSEA *= 0.051 [90% CI: 0.049–0.053]). The chi-square difference test (metric vs. configural model) was not significant (*χ^2^*_(__*10*__)_ = 13.255, *p* > .05), indicating that the same loadings were observed. The scalar model, assuming the same intercepts and thresholds, although demonstrated an acceptable model fit (*χ^2^*_(__*168*__)_ = 5,912.841, *p* < .001; *CFI *= 0.920; *TLI *= 0.913; *RMSEA *= 0.070 [90% CI: 0.068–0.072]), was not supported as the chi-square difference test (scalar vs. metrics) was significant. Given that the metric model was satisfied, we proceed to the multigroup structural model to test whether age moderates the links between financial capability and economic well-being.

Table 4 presents the results of multigroup path analyses with covariate control. We first estimated the SEM model separately for two age groups (M1a: aged 50–64; M1b: aged 65+), and the results suggested an acceptable model fit for both age groups (*CFI*: 0.922–0.929; *TLI*: 0.900–0.906; *RMSEA*: 0.052–0.053). We subsequently performed the multigroup structural invariance analysis, with the baseline model assuming the same factor loadings, but the structural paths were freely estimated (FL→FB, FA→FB, and FB→EW; i.e., M2), and the results suggested an acceptable model fit (*χ^2^*_(__*280*__)_ = 4,060.532, *p* < .001; *CFI *= 0.930; *TLI *= 0.912; *RMSEA *= 0.050 [90% CI: 0.048–0.051]). We then fixed the three paths to be equal across age groups, and the results were also acceptable and slightly better (*χ^2^*_(__*283*__)_ = 4,009.248, *p* < .001; *CFI *= 0.931; *TLI *= 0.914; *RMSEA *= 0.049 [90% CI: 0.048–0.051]). However, the model comparison using the chi-square test yielded insignificant results (*χ^2^*_(__*3*__)_ = 4.887, *p *> 0.05), suggesting that age had no moderating effect as the paths did not differ between those aged 50–64 and 65+.

**Table 4.** Multigroup SEM (MGSEM) analysis.

Model fit	MGSEM M1a (Aged 50–64)	MGSEM M1b (Aged 65+)	MGSEM M2	MGSEM M3
***χ^2^_(df)_***	2,362.715***_(132)_	1,868.499***_(132)_	4,060.532***_(280)_	4,009.248***_(283)_
***χ^2^* difference_*(df)*_**				4.887_(3)_
***CFI***	0.929	0.922	0.930	0.931
***TLI***	0.906	0.900	0.912	0.914
***RMSEA* [90% CI]**	0.052 [0.050, 0.054]	0.053 [0.051, 0.055]	0.050 [0.048, 0.051]	0.049 [0.048, 0.050]

*Note.* M1a = Aged 50–64 estimates only; M1b = Aged 65+ estimates only; M2 = multigroup analysis with paths (FL→FB, FA→FB, and FB→EW) were freely estimated by age groups; M3 = multigroup analysis with paths fixed across age groups; FL = financial literacy; FA = financial access; FB = financial behavior; EW = economic well-being; SE = standard error; CI = confidence interval; *CFI* = Comparative Fit Index; *TLI* = Tucker-Lewis Index; *RMSEA* = Root Mean Square Error of Approximation. All analyses were controlled for the covariates listed in Table 1.

*** *p* < .001.

### Sensitivity test

Although our theory-driven SEM models demonstrate satisfactory model fits, alternative or equivalent models may exist. We tested two alternative models: one assuming independent effects of financial capability on economic well-being (FL, FA, & FB→EW) and another assuming a sequential mediation effect (FL→FA→FB→EW). The results (see Supplementary Table 5) show that the models have poor or inestimable model fit. Therefore, our proposed model is considered acceptable for fitting the data well.

## Discussion

Using population-based data of older adults from the 2018 NFCS, this study confirms that financial capability is a multidimensional construct that is positively associated with economic well-being in later life. The measurement model supports that financial capability can be distinguished by financial literacy, access, and behavior, and the SEM results show that financial literacy and access are positively associated with financial behavior. Furthermore, the significant indirect effects show that financial behavior mediates the relationship between financial literacy, access, and economic well-being. However, the insignificant results of multigroup analysis show no differences in the paths, suggesting that age does not moderate these associations. Below, we discuss the implications and limitations of this study and future directions.

Financial capability can be conceptualized as comprising financial literacy, access, and behavior (Sherraden, 2013). However, measurement heterogeneity (e.g., single or combined measurement for financial capability) remains in the current evidence (Birkenmaier et al., 2022; Chen & Sun, 2023). Consistent with recent research (Chen & Sun, 2023; Fu, 2020; Sun & Chen, 2022), our study shows that financial capability is multidimensional but should be further distinguished by ability (financial literacy), opportunity (financial access), and action (financial behavior) (Sherraden, 2013). This measurement distinction echoes a recent scoping review (Birkenmaier et al., 2022), which calls for consistent measures of financial capability to facilitate comparison, standardization, and harmonization of findings across studies.

Findings also support the sequential relationship proposed by the financial capability framework, as financial literacy and access are positively correlated with financial behavior (H1 supported). Additionally, financial behavior mediates the relationships between financial literacy, access, and economic well-being (H2 supported). These findings contribute to the body of research examining the independent effects of financial capability when measured as separate constructs (Chen & Sun, 2023; Fu, 2020). Findings of significant indirect effects are similar to those of Sun et al. (2022); however, there are differences. Unlike the study by Sun et al. (2022), which showed that financial access is more critical in shaping financial behavior and reducing economic hardships among households aged 18 and above, we found that financial literacy and access operate similarly on financial behavior and economic well-being among older adults. These findings further support the notion that financial capability is context-specific, as the effects and associations may vary by individuals’ circumstances, such as age, gender, or race (Sherraden et al., 2018).

The similar effects of financial literacy and financial access on economic well-being via financial behavior have important implications for older adults. Current practices often prioritize educational programs that enhance the financial knowledge of older adults, such as focusing on retirement preparedness and preventing financial fraud (Nam & Loibl, 2021). Such an emphasis is largely driven by the extensive body of research on financial literacy (Finke et al., 2017; Gamble et al., 2015; Xiao et al., 2015). However, policies or programs that only target financial literacy overlook a critical aspect of financial capability. Our findings suggest that expanding financial access, promoting financial inclusion, and removing barriers to mainstream financial products for older adults are just as vital as strengthening financial literacy. Interventions that address only parts of financial capability may fail to adequately support financial stability and development later in life.

Although the bivariate analyses show that older adults (aged 65+) reported higher financial capability and economic well-being than their middle-aged counterparts (aged 50–64), these age differences are not reflected in the links between financial capability and economic well-being. The insignificant multigroup analyses suggest that the associations between financial capability and well-being are not moderated by age (H3 was not supported). However, such differences may result from variations in study design. Previous research has considered only age as a covariate. In contrast, our study examines whether and how the associations operate differently by age. The insignificant multigroup results suggest that the relationships between financial capability and economic well-being are similar across older and middle-aged adults.

The insignificant group comparison in the links between financial capability and economic well-being suggests that both age groups may have similar financial goals, such as retirement planning, managing retirement income, and ensuring long-term financial security (Gamble et al., 2015; Nam & Loibl, 2021). Such findings could be framed by the human agency concept of the life course perspective, as both middle-aged and older adults, although in different life stages, may share similar goals and actions for achieving financial security as they approach retirement. This may also reflect the fact that nearly half of older Americans believe they will not be financially prepared for retirement (National Council on Aging, 2024b). Furthermore, people concerned about having sufficient retirement income and financial stability may adopt more conservative financial behaviors and make more prudent financial decisions as they approach or enter retirement (Xue et al., 2021). The findings suggest that policies and programs should develop products and services that address common financial needs and specific financial challenges in later life, such as managing retirement income, healthcare costs, and expenses on long-term support and services, as well as ensuring access to appropriate and affordable financial products and services (Bennett et al., 2012; Chen & Sun, 2023).

It is important to acknowledge that inequitable outcomes in economic well-being persist. This highlights the need for using an equity lens in financial interventions, which focuses especially on populations struggling financially. Consistent with prior research (Kullgren et al., 2024), the results of the covariates on the latent variables of financial capability and economic well-being (Supplementary Table 3) showed that women and those with lower incomes reported lower economic well-being. We also find lower levels of economic well-being among White participants, and those with higher levels of educational attainment are associated with lower economic well-being. These findings suggest the need to examine the intertwined intersectionality of race and gender, as patterns may be obscured when gender and race are examined separately. Therefore, we conducted exploratory analyses to introduce the race-gender intersectional indicators as covariates (see Supplementary Table 6), extending the previous covariate control (Supplementary Table 3). Using White men as the reference group, the intersectional patterns are more nuanced. First, a clear gender-race intersectional gradient in financial literacy was observed: White men had the highest financial literacy, followed by men of color, White women, and women of color. Second, there were pronounced racial disparities in financial access, with men of color and women of color exhibiting lower financial access compared to White men. Third, significant gender effects were evident in financial behavior: White women and women of color scored higher than White men. Lastly, a salient gender difference in economic well-being among White participants: White women report lower economic well-being than White men.

Although this study was theorized and specified a priori to examine age-based heterogeneity in the pathways from financial capability to economic well-being, these significant educational, race, gender, and potential intersectional effects suggest varied expectations, experiences, and exposures regarding financial development and economic stability faced by women, racial/ethnic minorities, and especially women of color. The findings indicate that inequality in financial capability among socially constructed groups (based on gender, race, class, and others) may perpetuate socioeconomic disadvantages through differential access to education, financial services, and opportunities for financial development and asset building (Sherraden et al., 2018). Research has shown that women, minoritized racial and ethnic groups, immigrants, and low-income individuals are more likely to be excluded by mainstream financial sectors, face challenges in asset building, and have a higher reliance on high-cost credit options (e.g., payday loans) as a result of laws or institutions that intersect with structural discrimination against one’s socioeconomic status, gender, race, age, or country-of-origin (Birkenmaier & Fu, 2016; Sherraden et al., 2018). Therefore, policies or practices aimed at promoting financial capability but ignoring systemic and structural inequalities may produce ineffective interventions in redressing economic disparities (Holman & Walker, 2021; Homan et al., 2021). Furthermore, we urge future research to explore the role of expectations and how the influences of gender, race, class, and their intersectionality may be translated into the links between financial capability and economic well-being in later life.

Several limitations should be considered when interpreting the findings. First, we used the cross-sectional 2018 NFCS for data analysis as there was no existing panel data that collected comprehensive measures of financial capability. Although SEM can be applied to cross-sectional data, causal inference still requires strong theoretical justification (Hair et al., 2019). Guided by the financial capability framework, this study tests theory-driven models; however, its cross-sectional design limits causal claims, and the findings should be interpreted as associations rather than causal effects. The design also precludes conclusions about the development of financial capability over time. Accordingly, age comparisons reflect cross-sectional differences between age groups, not cohort or longitudinal effects. When available, longitudinal data should be used to re-examine the sequential relationships and age variations posited in the financial capability framework. Second, our analyses on older samples are unweighted, as weight only applies when analyzing the full sample of individuals aged 18 and older. Therefore, the generalizability of our findings to all older adults may be limited. Third, some objective financial literacy items (e.g., bonds, interest rates, stocks) may conflate financial knowledge with numeracy and thus attenuate or bias associations for respondents with lower computational ability. Nevertheless, the integration of both objective and subjective literacy items in this study may reduce reliance on any single operationalization of literacy. Lastly, the financial capability framework suggests that several structural, organizational, and interpersonal variables are important antecedents of financial capability, such as social and economic structure, financial socialization and guidance, and features of financial products (e.g., appropriateness, affordable, easy-to-use, etc). However, we were unable to include these variables in the model because they were not measured in the 2018 NFCS.

This study has implications for practice and policy. From the life course perspective, there are long-term lags for economic benefits to manifest through financial capability. Therefore, to bolster economic well-being later in life, practitioners should consider integrating policies and services to improve financial capability across people’s lives. Providing direct access to financial services, education, assessments, and guidance throughout people’s lives could establish a more solid economic foundation for old age (Chen & Sun, 2023; Sun et al., 2022). Specifically, these services should be tailored to meet later-life needs (e.g., managing retirement income, retirement planning, and budgeting) to achieve effective financial management. Regarding policy, financially inclusive policies should be developed to reach underserved groups, especially those with limited financial literacy and restricted access to financial services (Sherraden et al., 2024). Investments in financial technology and financial products with features that are available, affordable, accessible, appropriate, and easy to use are necessary to bridge the gaps in financial access. Lastly, social service professionals require training, education, and skill development in financial matters to provide financial guidance and resources to their constituents and to influence policies (Sherraden et al., 2024).

## Supplementary Material

igag003_Supplementary_Data

## Data Availability

This study was not preregistered. The National Financial Capability Study is publicly available, and public data files can be requested through the website: https://finrafoundation.org/nfcs-data-and-downloads
