# Supplementary material for: Financial capability and economic well-being of older Americans: empirical insights from the financial capability framework
Source: Innov Aging. 2026 Jan 10;10(4):igag003. doi: 10.1093/geroni/igag003 (PMC13031003; doi:10.1093/geroni/igag003)
Supplement: igag003_Supplementary_Data [file igag003_supplementary_data.docx]

***Innovation in Aging* Supplementary Material: Chen, Sun, Huang, & Sherraden. Financial capability and economic well-being of older Americans: Empirical insights from the financial capability framework.**

**Supplementary Table 1.** Characteristics of financial capability for all samples and comparison by age groups (aged 50–64 and aged 65+).

| **Financial capability** | ***M* (*SD*) or *N* (%)** | | | ***χ^2^ / t value*** |
| --- | --- | --- | --- | --- |
|  | **All  (*N*=12,840)** | **Age 50–64 (*n*=7,328)** | **Age 65+ (*n*=5,512)** |  |
| Financial literacy |  |  |  |  |
| Day-to-day financial matters (*range*: 1–7) | 6.13 (1.32) | 5.99 (1.38) | 6.32 (1.22) | *t*=−13.97*** |
| Self-rated financial knowledge (*range*: 1–7) | 5.37 (1.19) | 5.23 (1.24) | 5.55 (1.08) | *t*=−15.33*** |
| Objective financial literacy (*range*: 0–6)^a^ | 3.69 (1.51) | 3.57 (1.52) | 3.84 (1.48) | *t*=−10.01*** |
| Financial access |  |  |  |  |
| Checking accounts (*yes*) | 12,203 (96.40%) | 6,849 (94.87%) | 5,354 (98.42%) | *χ²*=112.24*** |
| Savings accounts (*yes*) | 10,222 (81.20%) | 5,588(77.83%) | 4,634 (85.69%) | *χ²*=124.88*** |
| Individual retirement accounts (*yes*) | 5,954 (48.38%) | 2,980 (42.51%) | 2,974 (56.13%) | *χ²*=224.26*** |
| Investment accounts (*yes*) | 5,204 (43.42%) | 2,598 (38.56%) | 2,606 (49.66%) | *χ²*=147.79*** |
| Have credit card(s) (*yes*) | 11,031 (87.49%) | 6,022 (83.72%) | 5,009 (92.50%) | *χ²*=217.72*** |
| Financial behavior |  |  |  |  |
| Set rainy-day fund (*yes*) | 7,697 (62.15%) | 3,876 (54.83%) | 3,821 (71.89%) | *χ²*=375.37*** |
| Spend less than or equal to household income (*yes*) | 10,747 (86.13%) | 6,024 (84.88%) | 4,723 (87.77%) | *χ²*=21.41*** |
| Economic well-being |  |  |  |  |
| Financial satisfaction (*range*: 1–10) | 6.27 (2.78) | 5.78 (2.83) | 6.93 (2.56) | *t*=−23.75*** |
| Retirement worry, reversed coded (*range*: 1–7) | 3.84 (2.08) | 3.46 (2.04) | 4.35 (2.03) | *t*=−24.28*** |
| Just getting by financially (*range*: 1–5) | 3.43 (1.41) | 3.23 (1.42) | 3.71 (1.34) | *t*=−19.17*** |
| Confidence in coming up with $2,000 (*range*: 1–4) | 3.24 (1.09) | 3.08 (1.15) | 3.44 (0.96) | *t*=−18.79*** |

*Note.* **p*<.05; ***p*<.01; *p*<.001. ^a^ The six objective literacy items are as follows: (1) Suppose you owe $1,000 on a loan and the interest rate is 20% per year compounded annually. If you didn’t pay anything off, at this interest rate, how many years would it take for the amount you owe to double? (Response: 1: *<2 years*, 2: *2-5 years*, 3: *5-10 years*, 4: *10 years+*). (2) Suppose you had $100 in a savings account and the interest rate was 2% per year. After 5 years, how much do you think you would have in the account if you left the money to grow? (Response: 1: *>$102*, 2: *Exactly $102*, 3: *< $102*). (3) Imagine that the interest rate on your savings account was 1% per year and inflation was 2% per year. After 1 year, how much would you be able to buy with the money in this account? (Response: 1: *More than today*, 2: *Exactly the same*, 3: *Less than today*). (4) If interest rates rise, what will typically happen to bond prices? (Response: 1: *They will rise*, 2: *They will fall*, 3: *They will stay the same*, 4: *These is no relationship between the two*). (5) A 15-year mortgage typically requires higher monthly payments than a 30-year mortgage, but the total interest paid over the life of the loan will be less. (Response: 1: *True*, 2: *False*). (6) Buying a single company’s stock usually provides a safer return than a stock mutual fund (Response: 1: *True*, 2: *False*)

**Supplementary Table 2.** Comparisons between unimputed and imputed results

|  | **Unimputed data** | **Imputed data** |
| --- | --- | --- |
| Direct effects (unstandardized *b*) |  |  |
| FL→FB | 0.810***  (0.072) | 0.812***  (0.059) |
| FA→FB | 0.696***  (0.065) | 0.696***  (0.049) |
| FB→EW | 1.838***  (0.038) | 1.807***  (0.035) |
| Direct effects (standardized *β*) |  |  |
| FL→FB | 0.486***  (0.037) | 0.483***  (0.029) |
| FA→FB | 0.454***  (0.033) | 0.460***  (0.026) |
| FB→EW | 0.879***  (0.012) | 0.875***  (0.010) |
| Model fits |  |  |
| *χ^2^ _(df)_* | 4206.128*** _(132)_ | 5007.963*** _(132)_ |
| CFI | 0.928 | 0.927 |
| TLI | 0.905 | 0.903 |
| RMSEA [90% *CI*] | 0.053 [0.052, 0.055] | 0.054 |

*Note.* FL=financial literacy; FA=financial access; FB=financial behavior; EW=economic well-being; SE=standard error; CI=confidence interval. The estimates were based on all respondents as no group differences were found via multigroup analysis; all the analyses were controlled for covariates in Table 1. The 90% *CI* of RMSEA for the imputed results was unavailable in multiple imputations (*n*=20). CFI = Comparative Fit Index. TLI = Tucker-Lewis Index. RMSEA = Root Mean Square Error of Approximation. ****p*<.001

**Supplementary Table 3.** Effects of covariates on latent factors of financial capability and economic well-being (unstandardized *b*).

|  | **Financial literacy**  **(FL)** | **Financial access**  **(FA)** | **Financial behavior**  **(FB)** | **Economic well-being**  **(EW)** |
| --- | --- | --- | --- | --- |
| Age (years) | 0.02***  (0.001) | 0.02***  (0.001) | 0.01  (0.01) | 0.02***  (0.003) |
| Women (*ref*: men) | −0.24***  (0.01) | −0.03*  (0.01) | 0.15***  (0.03) | −0.21***  (0.42) |
| White (*ref*: non-White) | 0.19***  (0.02) | 0.23***  (0.02) | −0.07*  (0.04) | −0.13*  (0.06) |
| Education | 0.07***  (0.01) | 0.06***  (0.01) | −0.07***  (0.01) | −0.04**  (0.01) |
| Household income | 0.14***  (0.01) | 0.21***  (0.01) | 0.03**  (0.01) | 0.14***  (0.02) |
| Received financial education | 0.19***  (0.03) | 0.05**  (0.02) |  |  |

*Note.* **p*<.05; ***p*<.01; *p*<.001.

**Supplementary Table 4.** Multigroup CFA (MGCFA) analysis

| **Model fit** | **MGCFA M1a**  **(Aged 50–64)** | **MGCFA M1b**  **(Aged 65+)** | **M2**  **(Configural)** | **M3**  **(Metrics)** | **M4**  **(Scalar)** |
| --- | --- | --- | --- | --- | --- |
| *χ^2^ _(df)_* | 2175.294*** _(71)_ | 1718.474*** _(71)_ | 3798.249*** _(142)_ | 2622.736*** _(152)_ | 5912.841***  _(168)_ |
| *∆χ^2^*  *_(df)_* |  |  |  | 13.255_(10)_ | 1873.550*** _(16)_ |
| CFI | 0.960 | 0.953 | 0.945 | 0.963 | 0.920 |
| TLI | 0.948 | 0.940 | 0.929 | 0.954 | 0.913 |
| RMSEA  [90% *CI*] | 0.064  [0.061, 0.066] | 0.065  [0.062, 0.068] | 0.063  [0.062, 0.065] | 0.051  [0.049, 0.053] | 0.070  [0.068, 0.072] |

*Note.* M1a: Aged 50–64 estimates only; M1b: Aged 65+ estimates only; M2: multigroup analysis test for configural model (i.e., same form); M3: multigroup analysis test for same factor loadings (i.e., same metrics); M3: multigroup analysis test for scalar model (i.e., same intercept and threshold). CI=confidence interval. CFI = Comparative Fit Index. TLI = Tucker-Lewis Index. RMSEA = Root Mean Square Error of Approximation. ****p*<.001

**Supplementary Table 5.** Alternative models

|  | **Model specification** | ***χ^2^ _(df)_*** | **CFI** | **TLI** | **RMSEA [90%CI]** |
| --- | --- | --- | --- | --- | --- |
| Proposed model | FL→FB  FA→FB  FB→EW | 4206.128*** _(132)_ | 0.928 | 0.905 | 0.053 [0.052, 0.055] |
| Alternative model 1 (A1) | FL→EW  FA→EW  FB→EW | 9234.334*** _(132)_ | 0.893 | 0.787 | 0.079 [0.078, 0.081] |
| Alternative model 2 (A2) | FL→FA  FA→FB  FB→EW | The model cannot be computed. | | | |

*Note.* FL=financial literacy; FA=financial access; FB=financial behavior; EW=economic well-being; CI=confidence interval. The estimates were based on all respondents as no group differences were found via multigroup analysis; all the analyses were controlled for covariates in Table 1. CFI = Comparative Fit Index. TLI = Tucker-Lewis Index. RMSEA = Root Mean Square Error of Approximation. ****p*<.001

**Supplementary Table 6.** Effects of gender-race intersectionality on latent factors of financial capability and economic well-being (unstandardized *b*).

|  | **Financial literacy**  **(FL)** | **Financial access**  **(FA)** | **Financial behavior**  **(FB)** | **Economic well-being**  **(EW)** |
| --- | --- | --- | --- | --- |
| Age (years) | 0.02***  (0.001) | 0.02***  (0.001) | 0.01  (0.01) | 0.02***  (0.003) |
| Race-gender intersectionality (*ref*: White men) | | | | |
| White women | −0.24***  (0.04) | −0.01  (0.01) | 0.15***  (0.03) | −0.22***  (0.04) |
| Men of color | −0.18***  (0.03) | −0.17***  (0.02) | 0.05  (0.05) | 0.09  (0.08) |
| Women of color | −0.41***  (0.02) | −0.27*  (0.03) | 0.23***  (0.05) | −0.07  (0.07) |
| Education | 0.07***  (0.01) | 0.07***  (0.01) | −0.07***  (0.01) | −0.04**  (0.01) |
| Household income | 0.13***  (0.01) | 0.21***  (0.01) | 0.03**  (0.01) | 0.14***  (0.02) |
| Received financial education | 0.19***  (0.03) | 0.05**  (0.02) |  |  |

*Note.* **p*<.05; ***p*<.01; *p*<.001.
